# Assessment of adherence to corticosteroids in asthma by drug monitoring or fractional exhaled nitric oxide: A literature review

**DOI:** 10.1111/cea.13787

**Published:** 2020-11-27

**Authors:** Fahad Alahmadi, Adam Peel, Brian Keevil, Rob Niven, Stephen J. Fowler

**Affiliations:** ^1^ Division of Infection, Immunity & Respiratory Medicine Faculty of Biology, Medicine and Health School of Biological Sciences The University of Manchester and Manchester Academic Health Science Centre and NIHR Manchester Biomedical Research Unit and Manchester University NHS Foundation Trust Manchester UK; ^2^ Respiratory Therapy Department College of Medical Rehabilitation Sciences Taibah University Madinah Saudi Arabia; ^3^ Norwich Medical School University of East Anglia Norwich UK

**Keywords:** adherence, asthma, corticosteroids, fractional exhaled nitric oxide

## Abstract

**Background:**

Although the efficacy of corticosteroid treatment in controlling asthma is widely accepted, its effectiveness is undermined by poor adherence. However, the optimal method for measuring adherence to asthma therapy remains unclear.

**Objective:**

To perform a review of the literature reporting biological, objective methods for assessing adherence to inhaled or oral corticosteroids in asthma; we included studies reporting direct measurement of exogenous corticosteroids in blood, or the effect of adherence on exhaled nitric oxide.

**Design:**

We searched three databases MEDLINE (using both PubMed and Ovid), the Cumulative Index of Nursing and Allied Health Literature (CINAHL) and Web of Science for articles published between January 1975 and July 2020. Quality of the studies was assessed using the National Institute of Health checklist.

**Results:**

From 2850 screened articles, 26 fulfilled the inclusion criteria. Measurement of blood prednisolone with or without cortisol was used in eight studies as a measure of oral corticosteroid adherence, and fractional exhaled nitric oxide (FeNO) in 17 studies for inhaled corticosteroid adherence. Inhaled corticosteroids were measured directly in the blood in one study. By direct measurement of drug levels in the blood, adherence rates to oral corticosteroids ranged from 47% to 92%, although the performance and timing of the test were often not known, making interpretation of findings and serum prednisolone concentrations difficult. FeNO is generally lower in adherent than non‐adherent patients, but no absolute cut‐off can be proposed based on the available data. However, a fall in FeNO following supervised inhaled corticosteroid dosing could indicate previous poor adherence.

**Conclusions and Clinical Relevance:**

Despite prednisolone and cortisol levels commonly being used as adherence markers in clinical practice, further work is required to assess the influence of the dose and timing on blood levels. The promising findings of FeNO suppression testing should be explored in further prospective studies.

## 1 INTRODUCTION

Asthma is the most common chronic inflammatory airway disease, affecting more than 300 million people worldwide.^1^ Inhaled corticosteroids (ICS) are the foundation of daily asthma control medication, and in severe asthma are needed at high doses, often in combination with daily oral corticosteroids (OCS).^2^ A high prevalence of poor adherence has been identified in all asthma severities, which impacts exacerbation and hospitalization rates, quality of life and pulmonary function. The impact on healthcare costs is complex, with non‐adherence resulting in lower direct spending on inhalers, but potentially higher costs associated with exacerbations, systemic steroid toxicity and escalation to biological therapy.^1^, ^3^, ^4^, ^5^, ^6^

Measuring adherence to asthma medications is recommended in national and international guidelines,^2^, ^7^ but represents a significant challenge. Methods of monitoring adherence should be accurate, easy‐to‐use and cost‐effective. They can be classified into objective and subjective methods, but all have drawbacks and none would be considered as a “gold standard.” The most common subjective methods of adherence include self‐report questionnaires, patient diaries, and assessment by healthcare providers. This is known to be unreliable, as patients particularly tend to overestimate their adherence.^3^ Objective assessments, which include pill counting, prescription pick‐ups and electronic device monitoring (smart inhalers), may also overestimate adherence where patients pick up or actuate their inhalers without inhaling the drug as prescribed. The effect of corticosteroid medications (pharmacodynamics) on blood biomarkers such as blood eosinophils^8^, ^9^, ^10^ or cortisol^11^ may be useful in ascertaining efficacy and toxicity, but this may not directly reflect adherence. The fractional exhaled nitric oxide test (FeNO) is an alternative biological non‐invasive method used as a surrogate marker of ICS adherence,^12^, ^13^, ^14^ due to the effect of ICS on nitric oxide synthase.^15^

Direct drug monitoring in biological fluids is probably considered the most reliable and accurate method, as it at least provides evidence that the patient has taken the medication sometime in the recent past, according to the specific assay and pharmacokinetics. Numerous studies have used blood assays to identify non‐adherence in asthma patients regarding prescribed daily OCS by measuring prednisolone and cortisol levels,^9^, ^16^, ^17^ and more recently, a similar approach has been reported for ICS.^18^

We have conducted a review aiming to identify and summarize the available literature on two objective biological methods that have been used to assess the adherence to either ICS or OCS in adults and children with asthma: (1) detection of inhaled or oral corticosteroids in body fluid, and (2) level of exhaled nitric oxide. Due to the heterogeneity of the studies within each adherence method included, a meta‐analysis was not conducted.

## 2 METHODS

### 2.1 Search strategy

This review was reported according to PRISMA (the Preferred Reporting Items for Systematic Reviews and Meta‐Analyses) guidelines.^19^ Although this was undertaken according to the standards of a systematic review in terms of search strategy, quality assessments and data extraction, PICO (Population, Intervention, Comparison and Outcome) reporting criteria were not suitable, due to multiple interventions of interest, and the lack of availability of a robust “gold standard” comparator for adherence in the vast majority of literature reviewed. The search was completed by 6 July 2020. The databases searched were MEDLINE (using both PubMed and Ovid), the Cumulative Index of Nursing and Allied Health Literature (CINAHL) and Web of Science. The terms used were (asthma OR asthmatic) AND (adherence OR compliance OR concordance) AND (oral corticosteroids OR inhaled corticosteroids) AND (exhaled nitric oxide OR FeNO). We screened the entire reference list from each eligible study, and Google Scholar was used to find any relevant citing articles. The study selection was performed based on the following inclusion and exclusion criteria.

### 2.2 Inclusion criteria

1. A clear diagnosis of asthma based on a physician's diagnosis or international guidelines.
2. Studies published in full, between January 1975 and July 2020
3. Primary research involving direct corticosteroid monitoring or the level of exhaled nitric oxide as a marker of adherence, or at least was investigated with an assessment of the rate of adherence.

### 2.3 Exclusion criteria

1. Other indirect methods of adherence (eg blood eosinophils or measures of adrenal suppression).
2. Non‐human studies.
3. Case series or case report.
4. Review articles.
5. Research in abstract form only.
6. Non–English‐language publications.

### 2.4 Study identification and data extraction

The first author screened all the titles and abstracts to exclude non‐relevant articles. All the selected studies were screened in full text to assess the eligibility. The last author (SF) confirmed the eligibility of studies and verified appropriate data extraction. Any disagreement between the authors in study selection and data extraction was resolved by discussion.

### 2.5 Quality assessment

We used the National Heart, Lung, and Blood Institute of the National Institutes of Health (NIH) quality assessment tools for observational, cohort and cross‐sectional studies, and controlled intervention studies.^20^ The first (FA) and the second (AP) reviewers evaluated the included studies individually.

### 2.6 Synthesis of results

Due to the different methodologies between the included studies, a meta‐analysis was not conducted.

## 3 RESULTS

**Figure 1:**
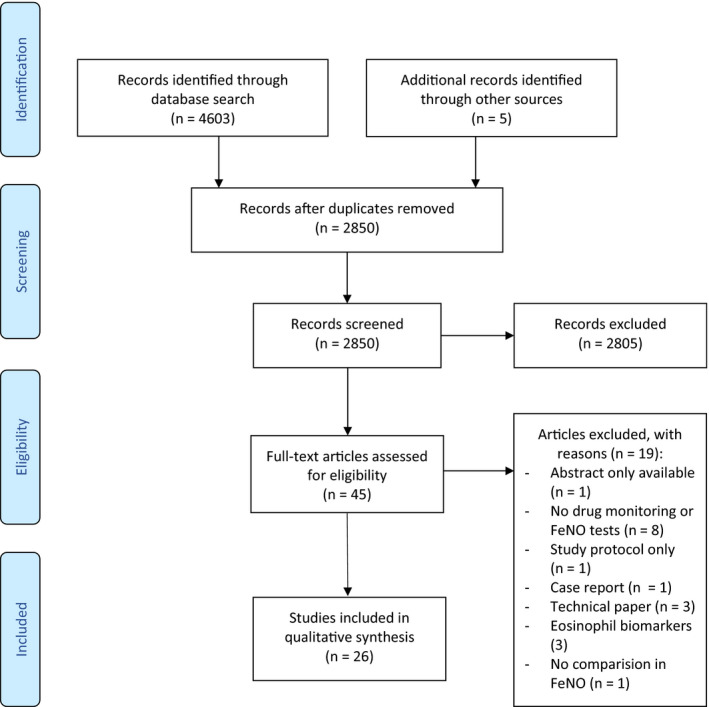
PRISMA flow diagram summary of study selection process

We identified 4603 studies through all databases. After removing duplicates, the titles and abstracts of 2850 articles were screened. The full texts of 40 potential articles were retrieved for further evaluation; 21 fulfilled the inclusion criteria. Reference list checking and screening Google Scholar added five studies, resulting in 26 studies included in total (Figure 1). Excluded studies are detailed in Table S1.

The relevant characteristics and findings of the selected articles are reported in Tables 1, 2, 3, 4, 5. The studies were conducted in 10 countries: 15 in the United Kingdom and Ireland, three in the Netherlands, two in the USA, and one each in Germany, Denmark, Spain, Finland, Greece and China. No relevant studies were found before 1998. The sample size of the included studies ranged from 17 to 529 participants. Thirteen studies included only children, 12 adults and only one both.

**Table 1:** Interventional studies reporting adherence to corticosteroids by direct serum measurement

First author, year	Participants^a^ and asthma severity (ICS dose)	Study description	Intervention, control and timing (where given)	Adherence measure and cut‐off	Adherence results
Payne et al. 2001^16^	*n* = 27, children Budesonide ≥1000 mcg/day or equivalent	Assessing adherence to prednisolone in patients treated with 2 weeks of OCS by measuring serum prednisolone and cortisol levels.	Oral prednisolone, 40 mg daily; no control The time between the last dose and OCS measurement was variable.	Serum prednisolone and cortisol level by HPLC. Good adherence: Last dose <24 h: detectable prednisolone + cortisol level <100 nM. Last dose >24 h: cortisol level <100 nM (no LoD given)	Two patients (2/27) had undetectable prednisolone or high cortisol levels. Prednisolone and cortisol levels not reported.
Payne et al. 2001^27^	*n* = 31, children Budesonide ≥1600 mc/day or equivalent	Assessing prednisolone adherence in patients treated with 2 weeks OCS by measuring serum prednisolone and cortisol levels.	Oral prednisolone, 40 mg daily; no control The time between the last dose and OCS measurement was variable.	Serum prednisolone and cortisol level by HPLC. Good adherence: Detectable prednisolone + cortisol level <100 nM (no LoD given)	Non‐adherence to prednisolone was identified in five patients. Prednisolone and cortisol levels not reported.
Lex et al. 2007^30^	*n* = 12, children and adolescents Budesonide ≥1600 mcg/day or equivalent	Assessing prednisolone adherence in patients treated with 2 weeks OCS by measuring serum prednisolone and cortisol levels.	Oral prednisolone, 40 mg daily; no control The time between the last dose and measurement was variable.	Serum prednisolone and cortisol level by HPLC. Good adherence: Detectable prednisolone + cortisol level <100 nM (no LoD given)	Three patients were considered as non‐adherent. Prednisolone and cortisol levels not reported.
Bossley et al. 2009^17^	*n* = 65, children Budesonide ≥800 mcg/day or equivalent µcg	Asthmatic patients referred to difficult asthma clinic; response (lung function, inflammation, symptoms) to corticosteroids (oral or IV) measured after 2 weeks. Adherence to OCS was assessed by the detection of prednisolone and cortisol in serum blood.	Oral prednisolone, 40 mg daily; no control The time between the last dose and measurement was variable.	Serum prednisolone and cortisol level; analytical method analysis not given Good adherence: Last dose <24 h: detectable prednisolone + cortisol level <100 nM. Last dose >24 h: cortisol level <100 nM (no LoD given)	12 patients (12/65) were non‐adherent. Prednisolone and cortisol levels not reported. Adherence was confirmed in all children who have a clinical corticosteroid response.

Abbreviations: HPLC, high‐performance liquid chromatography; ICS, inhaled corticosteroids; LoD, level of detection; OCS, oral corticosteroids.

^a^ We only considered those who have participated in the adherence measurements (not all the participants of the study).

**Table 2:** Studies reporting adherence to corticosteroids by direct serum drug measurements in patients on long‐term treatment

First author, year	Participants^a^ and asthma severity (ICS dose)	Study description	Corticosteroid route, dose, duration and timing (where given)	Adherence measure and cut‐off	Adherence results
Stirling, 1998^26^	*n* = 17, adult. BDP ≥2000 mcg/day or equivalent	Assessing the level of adherence to OCS by serum prednisolone level.	Levels measured 2 h after prednisolone dosing (dose not reported).	Serum prednisolone by HPLC. Good adherence: detectable prednisolone (no LoD given).	Prednisolone level in three patients (3/15) was undetectable. Mean (SD) levels 344 (285) nmol/L.
Robinson, 2003^28^	*n* = 18 “Confirmed asthma” *n* = 10 “unconfirmed asthma” BDP ≥1000 mcg/day or equivalent.	An observational study in uncontrolled patients on prednisolone attending the “difficult asthma” clinic.	Mean (range) dose of prednisolone in “confirmed asthma” 15 (12.5–125) mg/day. Mean (range) daily dose of prednisolone in “unconfirmed asthma” 30 (2–60) mg. Levels measured 2 h after prednisolone dosing (unsupervised).	Serum prednisolone and cortisol by HPLC. Good adherence: detectable prednisolone AND “normal” cortisol in patients on at least 15 mg/day (Reference range and LoD not given).	Confirmed asthma: nine patients “poorly adherent” (six with undetectable OCS and normal cortisol; three with detectable OCS and “normal” cortisol); mean (SD) prednisolone concentration (≥15 mg/day) 616 (143) mcg/L, and (<15 mg/day) 465 (90) mcg/L. Unconfirmed asthma: none were classed as non‐adherent; mean (SD) prednisolone concentration (≥15 mcg/day) 624 (125) mcg/L, and (<15 mg/day) 397 (136) mcg/L.
Gamble, 2009^9^	*n* = 51, adult Mean (SD) BDP equivalent 1388 (550) mcg/day.	Measuring the prevalence of non‐adherence to OCS among severe asthma and its relationship with asthma outcomes	Mean (SD) dose prednisolone 15.6 (11.1) mg/day. Levels measured 2–4 h after prednisolone dosing (unsupervised).	Serum prednisolone and cortisol (methods of analysis not given). Good OCS adherence: detectable prednisolone and undetectable cortisol (LoD not given; no reference given for cortisol level). Good ICS adherence: prescription refill ≥50%	Poor OCS adherence in 45% (23/51). Mean (SD) prednisolone level of the adherent group was 194 (160) ng/ml with undetected cortisol. Poor ICS adherence in 35% (8/23) Eight patients (8/26) who were adherent to OCS were filling <50% or fewer ICS prescriptions. Note: QoL score and hospital anxiety mean scores are higher in patients filling ≤50% in the preceding 12 months
George, 2017^18^	*n* = 19, adult. BDP ≥1000 mcg/day or equivalent.	Determining the feasibility of liquid chromatography‐mass spectrometry in detecting ICS over 8 h post‐dosing.	Inhaled fluticasone propionate dose range: 1000–1600 mcg; budesonide dose range: 400–800 mcg Directly observed and inhaler technique noted	Prescription refill for ICS in the last 6 months. ICS level measured by LC‐MS/MS. Good ICS adherence: prescription refill >80%; detectable ICS (LoD = 10 ng/ml)	18 subjects (18/19) had detectable serum ICS 8 h post‐dosing. ICS levels not reported. ICS adherence rate “undetectable baseline drug” in 52% (10/19).
Mansur, 2020^37^	*n* = 67, adult. BDP ≥1600/day or equivalent.	Evaluating the utility of liquid chromatography‐mass spectrometry in detecting the adherence to OCS.	Median (IQR) dose of prednisolone 10 (15) mg/day. Levels measured 5–8 h post‐dose (unsupervised)	OCS level measured by LC‐MS/MS. Good OCS adherence: Suppressed cortisol level by <100 nmol/L and detectable prednisolone (LoD = 20 nmol/L).	Cortisol suppressed in 40 subjects, from those 34 had detectable prednisolone levels (adherent), whilst undetectable prednisolone identified in the rest 6. Unsuppressed cortisol level with undetectable prednisolone was reported in 22 (non‐adherent). 5 subjects had prednisolone detected with unsuppressed cortisol. The median (IQR) of cortisol and prednisolone of the adherent group was 27 (48) nmol/L and 259 (622) nmol/L, respectively. In the non‐adherent group, it was 211 (130) nmol/L.

Abbreviations: BDP, beclomethasone dipropionate; HPLC, high‐performance liquid chromatography; ICS, inhaled corticosteroids; LC‐MS/MS, liquid chromatography‐tandem mass spectrometry; LoD, Level of detection; OCS, oral corticosteroids; QoL, quality of life; SD, standard deviation.

^a^ We only considered those who participated in the adherence measurements (not all the participants of the study).

**Table 3:** Changes in FeNO (cross‐sectional) in observational studies monitoring adherence by other methods

First author, year	Participants^a^ and asthma severity (ICS dose)	Brief study description	Adherence measure and cut‐off	Adherence results
Cano, 2010^31^	*n* = 67, children. Budesonide 200‐ 800 mcg/day or equivalent.	Identifying the main determinants of airway inflammation.	Self‐reported by patient or parents. Good adherence: if over 75% of the prescribed doses taken.	Good adherence rate: 83%. Lower FeNO with ICS versus no ICS [median (IQR) 27.5 (15.0–51.5) versus 41.8 (19.9–62.5) ppb]. FENO independently predicted adherence to ICS.
Scott, 2010^32^	*n* = 65, adult. ICS dose not reported.	Assessing the effect of atopy on FeNO.	Self‐reported. Good adherence: if participants reported forgetting ICS once a week or less.	Good adherence rate: 50% (30/59). No difference between atopic and non‐atopic asthma adherence versus good adherence.
Koster, 2011^22^	*n* = 527, children. ICS dose not reported.	Investigating factors affecting adherence.	MARS questionnaire completed by parent. Good adherence: cut‐off ≥21.	Good adherence rate: 57%. Factors associated with good adherence: low FeNO and young age.
Vijverber, 2012^23^	*n* = 529 children. ICS dose not reported.	Assessing FeNO as a marker of uncontrolled asthma and adherence.	MARS questionnaire completed by parent. Good adherence: cut‐off ≥21.	No overall adherence rate reported. Children with FeNO >25 ppb had lower adherence. FeNO measurements not reported for both groups (adherent and non‐adherent).
Klok, 2016^34^	*n* = 60, children. Low‐moderate fluticasone propionate.	FeNO level assessed as a marker for ICS adherence.	Smartinhaler‐recorded dosages Good adherence: If over 80% of the prescribed doses taken.	Overall median (IQR) adherence: 82 (56–90) %. Low FeNO associated with good adherence. High FeNO (>25 ppb) predicted poor adherence with a sensitivity of 41% and specificity of 94% when ICS adherence <80%.
Yuan, 2019^39^	*n* = 222, adult ICS (dose not reported)	Assessing the association between asthma exacerbations in 12 months of follow‐up and baseline FeNO.	Prescription records Good adherence: if >80% of the prescribed dose taken.	Good adherence rate: 53% Patient without exacerbations had better adherence rate (*p* = .004). No difference in the mean (IQR) of FeNO levels between good versus poor adherence groups: 25 (10–47) versus 29 (12–63) ppb.
Vähätalo, 2020^40^	*N* = 181, adult Budesonide equivalent 400–1000 mcg/day.	Assessing the variability of long‐term adherence within 12‐year follow‐up.	Prescription record (checking both prescribed and dispensed) Cut‐point of 80% was used to determine good adherence for both 12‐year adherence and annual adherence.	The average adherence rate over 12 years was 69%. Only 9% of patients had good annual adherence rate during the 12 year. 37% of patient had at least one period of non‐adherence during the 12 year. No difference was found in FeNO between the good and poor adherence groups.

Abbreviations: FeNO, fractional exhaled nitric oxide; ICS, inhaled corticosteroids; IQR, interquartile range; MARS, medication adherence response scale.

^a^ We only considered those who have participated in the adherence measurements (not the whole participants of the study).

**Table 4:** Repeated FeNO measurements in studies monitoring adherence by other methods

First author, year	Participants^a^ and asthma severity (ICS dose)	Brief study description	Adherence measures and cut‐off	Adherence results
Beck‐Ripp, 2002^12^	*n* = 15, children. Budesonide 200–400 mcg/day	Measuring FeNO to determine whether it could be used as a potential marker of inflammation	Dose‐counting, calculated as doses are taken/doses prescribed × 100 (%). Good adherence: If over >65% of the prescribed doses taken.	Mean (SD) FeNO pre‐ICS 14.0 (1.2) ppb; post‐ICS 7.7 (2.5) ppb. Positive correlation between ICS adherence and % reduction in FeNO (*r* ^2^ = .59; *p* = .0003). Adherence rate after 8 weeks of ICS not reported.
Claudia, 2004^29^	*n* = 30, Children ICS dose estimated range (84–2000) mcg BDP equivalent	To determine the utility of FeNO in assessing asthma control and ICS adherence.	Diaries (number of doses per day and number of days used as prescribed) then recorded as a percentage. Good adherence: if self‐reported adherence was >75% of their prescribed regime.	Poorly adherent patients had high FeNO compared with adherent patients (130 vs. 34 ppb, *p* = .001) Negative correlation between FeNO and adherence (*r* = −.76, *p* = .001).
Katsara, 2006^21^	*n* = 20, children ICS (dose not reported)	Assessing the relationship between FeNO levels and ICS compliance in asthmatic children.	A data logger was attached to the inhaler (recorded time and date of all actuations) Good Adherence: day compliance >60%.	No correlation between adherence and mean FeNO (*r* = .05, *p* = .67). No overall adherence rate reported. No repeated FeNO measurements reported
Szefler, 2008^35^	*n* = 276, adolescent and adult. Fluticasone dose range: 100–500 mcg/day	Determine whether FeNO could increase the effectiveness of asthma treatment	Dose counter. Good adherence: if 50% of the prescribed dose taken.	Mean (SD) adherence rate in the study was 89 (28) %. FENO lower in the adherent group (geometric mean 23.9 vs. 30.8).
Strandbygaard, 2010^36^	*n* = 26, adult. Fluticasone ICS (dose not reported) GINA 2 (*n* = 8) GINA 3 (*n* = 16) GINA 4 (*n* = 2)	The impact of daily SMS text message reminder on asthma treatment and change of FeNO over 12 week period	Dose counter. Good adherence: Dose count on Seretide Diskus within week 4 until week 12. Pharmacy record (cut‐off not reported)	In the intervention group, adherence rate did not change; in the control group, adherence decreased from 84% to 70%. FeNO fell in both groups. With no between‐group difference Data on pharmacy records not given.
Price, 2013^33^	*n* = 226, adult and children. BDP 100–400 mcg/day or equivalent	Evaluating FeNO in predicting steroid responsiveness.	Medication possession ratio (Defined as a number of days' supply of therapy/number of days in the total prescribing period × 100%) Good adherence: Unknown	Low baseline adherence to ICS associated with high FeNO (adult > 50 ppb, children > 35 ppb) In the following year, participants with high FeNo had improved adherence.
Jochmann, 2017^24^	*n* = 93, children and adolescents ICS dose range: 100–3200 mcg BDP/day or equivalent	Electronic ICS monitoring devices in identifying non‐adherence.	Smart inhaler attached to ICS Good adherence: if ≥80% of the prescribed dose taken. MARS‐5 (parent‐reported). Good adherence (MARS): Unknown	39/93 (42%) participants were adherent. Baseline visit: no difference between adherent and non‐adherent groups in FeNO. Improvement in FeNO in all participants at the end of monitoring (34 vs. 21 ppb; *p* = .001). Patients with good adherence and asthma control test >20 showed a significant reduction in FeNO in the follow‐up visit (median: 18 vs. 11 ppb). No correlation identified between MARS questionnaire and adherence.
Koumpagioti, 2020^38^	*n* = 39, children and adolescent. ICS (dose not reported)	To investigate the impact of asthma care educational programme on adherence.	Smart inhalers attached to ICS. Adherence was categorized as good (≥80%), moderate (60%–79%), poor (<60%).	Median (IQR) adherence rate 80 (73–85)% for the interventional group, with 51% of those reported as good adherers. Overall significant improvement in FeNO levels after ICS administration in interventional and non‐interventional groups.

Abbreviations: BDP, beclomethasone dipropionate; FeNO, fractional exhaled nitric oxide; ICS, inhaled corticosteroids; MARS, medication adherence response scale; SD, standard deviation.

^a^ We only considered those who have participated in the adherence measurements (not the whole participants of the study).

**Table 5:** FeNO as marker of adherence (cross‐sectional and suppression studies)

First author, year	Participants^a^ and asthma severity (ICS dose)	Brief study description	Adherence measure and cut‐off	Adherence results
McNicholl, 2012^14^	*n* = 146, adult. BDP 800–2000 mcg/day equivalent.	Assessing daily level of high FeNO suppression after using high dose of ICS for 7 days as marker in identifying non‐adherence.	Good ICS adherence: prescription refill ≤80% ICS. Good OCS adherence: detectable prednisolone and undetectable cortisol (LoD not given; no reference given for cortisol level).	At baseline: no correlation between baseline FeNO level and adherence; poor adherence rate: 76%. Five days of observed ICS: 13/40 of the participants were non‐adherent. 42% fall in FENO over 5 days of treatment with high‐dose ICS indicated a previous non‐adherence. Seven days of observed ICS: the non‐adherent group (*n* = 13) had a reduction in FeNO compared with the adherent group (*p* = .003).
Heaney, 2018^25^	*n* = 290, adult BDP 800–2000 mcg/day equivalent. OCS (*n* = 129)	The utility of FeNO suppression testing in clinical care using remote monitoring technologies.	Diskus inhaler connected with INCA device. Good adherence: defined as ≥70% FeNO levels: reduction by 42% from baseline FeNO level day 7 (positive FeNO suppression test).	FeNO after 7 days: Positive FeNO suppression identified in 130 patients [median FeNO = 88 ppb (64–127) vs. 32 ppb (21–46)] 45 (63%) patients from the negative FeNO suppression group (*n* = 70) on daily prednisolone. FeNO after 4 weeks: Positive FeNO suppression group (*n* = 85); good adherence rate: 63% Negative FeNo suppression group (*n* = 40); good adherence rate: 67% FeNO level at 7 days associated with FeNO after 4 weeks in good adherence group

Abbreviations: BDP, beclomethasone dipropionate; FeNO, fractional exhaled nitric oxide; ICS, inhaled corticosteroid; INCA, INhaler Compliance Assessment; IQR, interquartile range; OCS, oral corticosteroids.

^a^ We only considered those who have participated in the adherence measurements (not the whole participants of the study).

### 3.1 Quality assessment

Cohort and cross‐sectional designs were most common (*n* = 22); the remaining four studies were randomized controlled trials (RCTs). Generally, most of the studies (*n* = 14) were ranked as poor quality (see Tables S2 and S3). Of the observational cohort and cross‐sectional studies, seven were rated fair,^9^, ^14^, ^21^, ^22^, ^23^, ^39^, ^40^ three good^24^, ^25^, ^37^ and 12 poor‐quality.^16^, ^17^, ^18^, ^34^ Among the randomized controlled studies, one was rated as good,^35^ one fair^38^ and two poor.^12^, ^36^

### 3.2 Adherence methods

Oral corticosteroid adherence was assessed by direct measurement of blood levels in eight studies,^9^, ^16^, ^17^, ^26^, ^27^, ^28^, ^30^, ^37^ and ICS levels in one^18^ (Tables 1 and 2). Seventeen studies^12^, ^39^ used the FeNO test to indirectly assess adherence (Tables 3, 4, 5).

### 3.3 Corticosteroid adherence: assessment by direct monitoring of drug levels

#### 3.3.1 Methods used for corticosteroid detection

Oral prednisolone was the only OCS used among the included studies, which typically reported blood prednisolone level in addition to cortisol,^9^, ^16^, ^17^, ^27^, ^28^, ^30^, ^37^ applying various cut‐offs to each as shown in Tables 1 and 2. One study reported ICS levels over 8 h after witnessed inhalation.^18^ The analyses of prednisolone and cortisol concentrations were done by either high‐performance liquid chromatography (HPLC) or liquid chromatography‐mass spectrometry (LC‐MS), although important analytical parameters such as the limits of detection (LoD) or quantification (LoQ) were not mentioned in all but one,^37^ as shown in Tables 1 and 2. George et al.^18^ reported serum ICS levels measured using liquid chromatography‐tandem mass spectrometry (LC‐MS/MS) with a LoQ of 25 ng/L.

#### 3.3.2 Adherence rates by direct corticosteroid detection

Four studies investigated a daily dose of 40 mg prednisolone given as an “interventional” drug (Table 1). Overall poor adherence rates were between 8% and 25%.^16^, ^17^, ^27^, ^30^ Four further studies reported “real‐world adherence” in patients who were prescribed regular daily prednisolone at enrolment.^9^, ^26^, ^28^, ^37^ Stirling et al.^26^ reported three of 15 (20%) participants had undetectable prednisolone in the blood 2 h after dosing, although the daily prescribed dose was not reported. Mean (SD) prednisolone levels were 344 (285) nmol/L (approximately equivalent to 124 mcg/L). Robinson et al. assessed adherence in patients referred with “difficult” asthma and compared adherence rates between those with and without a subsequently confirmed diagnosis. They found that half of the participants (9/18) with confirmed asthma were non‐adherent, whilst none of the 10 patients in the non‐asthma group were considered as having poor adherence.^28^ Mean (SD) prednisolone concentration in the adherent patients prescribed at least 15 mg/day was 616 (143) mcg/L, and 465 (90) mcg/L in those prescribed <15 mg/day. In a cross‐sectional study in Northern Ireland, the prevalence of poor adherence to OCS was 23/49 (47%). Of those with undetected prednisolone, 35% had a good level of adherence based on ICS prescription refill >80% in the last 6 months.^9^ In this study, mean (SD) prednisolone levels of the adherent group (mean daily dose 15.6 mg) were 194 (160) mcg/L. Mansur et al. measured serum prednisolone and cortisol, estimated 5–8 h post‐prednisolone dosing (unwitnessed) in severe asthmatics. Good adherence (defined as suppressed morning cortisol) was identified in 40 of 67 subjects; median (IQR) serum cortisol was 27 (48) nmol/L and 259 (622) nmol/L for serum prednisolone.

In an ICS study,^18^ all participants were witnessed using their inhaler and had inhaler technique rated. One patient observed to have a poor inhaler technique did not have a detectable level of ICS. Eighteen of 19 (95%) participants had detectable ICS (budesonide or fluticasone propionate) in the serum 8 h after witnessed inhalation.

### 3.4 Adherence to inhaled corticosteroids and exhaled nitric oxide

There was marked heterogeneity in the 17 studies that measured FeNO levels. Three different designs were used: (1) studies investigating the association between a single FeNO measurement and ICS adherence (*n* = 7); (2) studies examining the association between repeated FeNO measurements and ICS adherence (*n* = 8); and (3) studies using the “FeNO suppression test” as a surrogate marker of adherence in patients using ICS (*n* = 2). All studies used the FeNO test accompanied by different objective adherence methods, for example prescription refill, pill count for a specific period or self‐reported questionnaires.

#### 3.4.1 Single FeNO test and adherence

Seven studies measured FeNO level at a single time‐point and reported FeNO in adherent and non‐adherent patients.^22^, ^23^, ^31^, ^32^, ^34^, ^39^, ^40^ Methods such as self‐reported questionnaires, electronic devices and patients' diaries were used in assessing the adherence rate. ICS dose and route were not controlled (ie participants used their usual medication). Cano et al.^31^ considered adherence satisfactory if parents reported that at least 75% of prescribed doses were taken, and found that adherence was the only independent predictor of FeNO in patients treated with ICS after adjusting for age, gender, BMI and ICS dose. Scott et al.^32^ defined poor adherence where the patient missed at least two doses of ICS per week; they found no difference in FeNO by adherence levels. A validated medication adherence self‐report scale (MARS), reported by the parents of participants, was used to identify poor adherence to ICS in two studies.^22^, ^23^ Both of the studies reported an association between adherence and FeNO. Koster et al.^22^ noted that FeNO was lower in adherent compared with non‐adherent patients. Vijverberg et al.^23^ investigated several confounding factors for FeNO, and found that adherence was an independent factor in patients with high FeNO (>25 ppb). One study evaluated adherence over 6–12 months by smart inhalers and reported an inverse correlation between adherence and FeNO (*r* = −.41; *p* = .001).^34^ Two recent studies used prescription records with ≥80% cut‐off point for good adherence in adult patients with asthma,^39^, ^40^ both found no difference in FeNO levels between groups with good and poor adherence.

#### 3.4.2 Repeated FeNO measurements and adherence

Longitudinal FeNO measurements and adherence were reported in eight studies conducted over 6 weeks to 12 months.^12^, ^21^, ^24^, ^29^, ^33^, ^35^, ^36^, ^38^ Beck‐Ripp et al.^12^ measured FeNO three times over 16 weeks and compared with estimated adherence by dose‐counting. Any patients with a history of suboptimal adherence or poor inhaler technique were excluded. In the first 4 weeks, children were prescribed high‐dose inhaled budesonide, which was then withheld between the fifth and the eighth week (the washout period). In the last 8 weeks, patients were randomized to 200 mcg daily inhaled budesonide versus no ICS and a correlation was identified (*r* ^2^ = .59, *p* = .0003) between ICS dose counts and percentage reduction in FeNO. Another study measured FeNO in 30 children and evaluated adherence by checking the diaries of the participants (number of daily doses missed) in each visit for 6 months.^29^ FeNO levels were significantly lower in those regularly using ICS versus the poorly adherent group (34 vs. 130 ppb, *p* = .001).

Similarly, in 2006, children used a data logger attached to their pMDI for 1 month and attended four visits to test the level of FeNO in each visit,^21^ but no link was demonstrated. Strandbygaard et al.^36^ reported data from pharmacy records alongside FeNO for 12 weeks and randomized patients to short message services (SMS) daily intervention reminder, or control (no intervention). However, despite the non‐SMS group having poorer adherence over 12 weeks, both groups of patients (SMS and non‐SMS) had significant improvements in FeNO. A large cohort study in adults and children found that those with a high FeNO had poor adherence by measuring the medication possession ratio (MPR) at baseline, but that this improved at 12 months.^33^ One good‐quality RCT compared the FeNO level with adherence. Adherence was evaluated at each visit by counting the missed doses in a Diskus device, and adherence was considered poor if <50% was taken during the treatment trial.^35^ A recent longitudinal observational study assessed the level of adherence in children by attaching an electronic monitoring device (Smartinhaler) to the participants' inhalers and by MARS questionnaire.^24^ There was an improvement in FeNO at the end of the trial in all participants, but no difference between the good and poorly adherent groups. Likewise, Koumpagioti et al.^38^ have used smart inhalers and found a general improvement in FeNO levels in both interventional and non‐interventional groups after 6 weeks of ICS; comparative FeNO levels between patients with good versus poor adherence were not reported.

### 3.5 FeNO suppression test as a marker of adherence

Two studies reported an application for FeNO testing in assessing adherence to ICS in patients with severe or uncontrolled asthma.^14^, ^25^ This method measures the daily level of FeNO along with direct observation or monitoring of participants using a high daily dose of ICS over 5–7 days, or 1 month. McNicholl et al.^14^ first reported the feasibility of using the FeNO suppression test to predict non‐adherence in patients with “difficult asthma” and high FeNO levels (≥45 ppb). They determined a positive FeNO suppression test where the FeNO level dropped beyond a threshold (log_10_ ΔFeNO ≥ 0.24), calculated by change from mean log_10_ FeNO (day 0–1) to mean log_10_ FeNO (day 6–7). This threshold demonstrated 78% sensitivity and 92% specificity for non‐adherence, determined by ICS prescription refill rates over the previous 6 months (adherent if >80%). They noted a high reduction of FeNO levels among the poorly adherent group (<70% of prescription refill), and the mean level of FeNO suppressed from 79 to 47 ppb (*p* = .003) after 7 days of observed ICS. Likewise, Heaney et al.^25^ used the FeNO suppression test and the same threshold for FeNO suppression testing over 7 days and 4 weeks and monitored adherence by a smart inhaler device attached to Diskus inhaler, which recorded the sound of the inhaler opening alongside the date, time and technique. FeNO in 65% (130/201) of the participants was suppressed (>42% reduction from baseline) after 1 week of ICS. However, data from almost 40% of participants were excluded, due to them either forgetting to measure daily FeNO or missing daily ICS doses.

## 4 DISCUSSION

We have reviewed studies using objective biological methods for adherence monitoring in asthma, specifically through drug concentration assays in blood or measurement of exhaled nitric oxide. Our key findings are as follows: (1) measurement of blood prednisolone levels, with or without serum cortisol, is commonly used as an adherence measure in studies, and if accurate, it suggests adherence to oral prednisolone is often poor, even within clinical trials; (2) whilst exhaled nitric oxide typically falls with inhaled corticosteroid use and is generally found to be lower in adherent versus non‐adherent patients, there appears to be no reliable cut‐off that can be used to classify adherence; (3) it may be that a fall in FeNO following monitored ICS use can identify previous non‐adherence, although this needs to be prospectively validated.

Although prednisolone detection was commonly used as marker of adherence, sometimes with simultaneous measurement of cortisol, none of the included papers cite data that support this assumption over the range of prednisolone doses used, and the timing of the sample post‐dose. Indeed, many studies did not report the dose or timing. Although prednisolone can likely be reliably detected for at least 8 h following a 40 mg dose,^41^ this cannot be assumed to hold for the lower doses that are commonly used for maintenance in severe asthma.^42^ If this measure is to be used in clinical practice, further dose‐ranging pharmacokinetic studies are required in this patient group. One study reporting the level of ICS within an 8‐h window in serum as a marker of adherence^18^ found that they could be detected in all but one of 19 patients. Whilst promising, this study was performed in patients taking only budesonide or fluticasone, and similar studies are needed for other commonly used ICS. Corticosteroids in biological samples were typically detected by either LC‐MS or HPLC. The mass analytical detector in LC‐MS gives a much higher sensitivity (~ about 1000‐fold) with a LoD in the low ng/ml range,^43^, ^44^ although it is relatively high cost and requires a skilled operator. In many of the included studies, we found that key methodological details and performance parameters, such as LoD or LoQ, were not given, thus making the application and generalizability of the findings difficult.

Exogenous corticosteroids have an adrenal suppressive effect,^16^, ^45^ and cortisol level was reported in several studies as a surrogate for adherence to oral corticosteroids,^9^, ^16^, ^17^, ^27^, ^28^, ^30^ although using varied and non‐validated cut‐offs. Many factors can influence cortisol levels, such as circadian rhythm, ICS use and the variability of cortisol values between individuals,^46^, ^47^ and so (as for prednisolone levels above) studies are required to validate timings and cut‐offs to support their clinical use in patients with severe asthma.

Most of these studies reporting direct serum monitoring were of poor or fair quality, with small sample sizes and cross‐sectional designs. Prednisolone administration was almost always unsupervised, and so it is unknown whether lack of detection represents non‐adherence or failure of the test. As expected, low adherence rates were more common in real‐world study designs (20%–50%) when compared to interventional studies (8%–25%). Whilst most guidelines acknowledge that adherence should be assessed, they do not suggest a cut‐off for “good” adherence. A cut‐off of 80% has been suggested for example by McNichol et al.^14^ and adopted widely (in the UK at least) as the standard required before escalation to biologics, with little supporting evidence. It may be that some patients “self‐titrate” their treatment to maintain good control, and apparent “low adherence” may not be very important in such cases. However when a patient with asthma presents with an exacerbation or poor control, the investigation must include an appraisal of whether the current treatment plan is working, and for that of course, we need to know whether it is being followed.

Most studies reporting a single measurement of FeNO,^22^, ^23^, ^31^, ^34^ or repeated measures,^12^, ^29^, ^33^, ^35^ found higher levels in poorly adherent subjects. There are not enough data available to be able to propose a single cut‐off, or a clinically meaningful change (fall) over time, for clinical use. This may be in part because important confounding factors such as age, height, gender and atopy were not taken into consideration.^48^ In addition, the “gold standard” adherence in these studies often had significant limitations. A number of studies measured the level of adherence by diaries or self‐report questionnaires.^22^, ^23^, ^29^, ^31^, ^32^ The accuracy and reliability of such methods are low,^49^ with patients typically over‐estimating their adherence.^5^, ^50^, ^51^ The FeNO suppression test may be able to identify patients with previous poor adherence,^14^ and may be useful in a clinical setting at least in patients with elevated FeNO levels (>45 ppb),^14^, ^25^ where adequate resources are available. Further, non‐suppressors may be identified with relative corticosteroid insensitivity who may require alternative therapeutic strategies.

Given the costs and time associated with adherence monitoring in asthma, it may be worth considering applying these methods only in selected patients where financial and clinical value will be most likely. The most obvious area would be in those with apparent severe disease who are being considered for biologics, where better ICS adherence may improve disease control without the need for such high‐cost therapies. In such patients, a relatively comprehensive assessment including pick‐up checking, direct monitoring (blood ICS/OCS levels), smart inhalers (when available) and FeNO suppression (where relevant) may be justified. Conversely, it may also prove useful at start of asthma therapy, where guidelines suggest a trial of ICS therapy as part of the diagnostic workup^2^; interpreting response without any knowledge of adherence is impossible and may lead to underdiagnoses or inappropriate therapy escalation (where there appears to be no response, but in fact, enough ICS has not been taken). It is here that the possibilities raised by the emergence of smart inhalers offer promise both in helping the clinician assess adherence patterns and potentially in helping the patient to establish adherent behaviours.

Several limitations of this review need to be considered. The included studies were selected based on the method of adherence from either direct drug measurement or measuring the FeNO level, and these methods cannot be compared with each other. Most studies were of low quality with high heterogeneity across the studies, including patient characteristics (in particular, asthma severity and age) and cut‐off values of both adherence methods. Methods not included in the review, but of potential use, include the detection of corticosteroid metabolites in the urine, as reported in Ref.^52^ In this study, fluticasone propionate‐17beta‐carboxylic acid was found in the urine of all 30 included subjects 16–24 h after witnessed inhalation of fluticasone propionate. A further single case report also found that fluticasone propionate and beclomethasone dipropionate were detectable in an induced sputum sample.^53^ Further improvements to methods that assess the hypothalamic‐pituitary‐adrenal (HPA) axis may also prove useful; Smy et al.^54^ found that hair cortisol during ICS treatment was reduced, and proposed that this as a useful surrogate marker of adherence. Finally, the robustness of this review would have been improved by independent searching, study selection and data extraction involving at least two researchers; here, one author performed these duties, and the results of each were subsequently verified by a second author.

In conclusion, we have reviewed the use of serum exogenous corticosteroid and cortisol detection, and of FeNO, as biological measures of adherence in asthma. Further work is required to adequately define the influence of drug dose‐ and timing‐dependent factors on prednisolone and cortisol levels, in order to support their use in clinical and research practice. Although FeNO is usually lower in adherent patients, there are no data available to allow a single cut‐off to be proposed. FeNO suppression testing merits further investigation as a marker of both adherence and steroid insensitivity.

## CONFLICT OF INTEREST

The authors declare no conflict of interest.

## AUTHOR CONTRIBUTION

FA, SJF, BK and RN conceived and designed the analysis, and drafted the manuscript; FA and AP performed the searches and assessed data quality; all authors revised the draft manuscript and approved the final accepted version.

## Supporting information

Table S1‐S3

## Data Availability

Data sharing is not applicable to this article as no new data were created or analysed in this study.
