# Supplementary material for: Assessment of adherence to corticosteroids in asthma by drug monitoring or fractional exhaled nitric oxide: A literature review
Source: Clin Exp Allergy. 2020 Nov 27;51(1):49–62. doi: 10.1111/cea.13787 (PMC7839457; doi:10.1111/cea.13787)
Supplement: Supplementary file 1 — Table S1‐S3 [file CEA-51-49-s001.docx]

Online Supplementary Material

**Assessment of adherence to corticosteroids in asthma by drug monitoring or fractional exhaled nitric oxide: a literature review.**

Fahad Alahmadi^1,2^, Adam Peel^3^, Brian Keevil^1^, Rob Niven^1^, Stephen J Fowler^1^

# Faculty of Biology, Medicine and Health, School of Biological Sciences, Division of Infection, Immunity & Respiratory Medicine, The University of Manchester and Manchester Academic Health Science Centre and NIHR Manchester Biomedical Research Unit and Manchester University NHS Foundation Trust, UK

# Respiratory Therapy Department, College of Medical Rehabilitation Sciences, Taibah University, Madinah, Saudi Arabia

# Norwich Medical School, University of East Anglia, UK

Table S1: Excluded Studies

| Ref | Author | Title | Reason |
| --- | --- | --- | --- |
| 1 | Knoery, 2015 | Non-adherence to inhaled corticosteroid therapy as an explanation for persistently elevated exhaled nitric oxide levels in patients with asthma: Effect of directly observed therapy with inhaled corticosteroids (DOT-ICS) | Abstract only |
| 2 | Agache, 2012 | Predictive Value of Lung Function Trend and FeNO for Difficult Asthma in Children | No direct measurement or not mentioning any correlation between FeNO and adherence level |
| 3 | Hederos, 2009 | Six-year follow-up of an intervention to improve the management of preschool children with asthma | No direct measurement or not mentioning any correlation between FeNO and adherence level |
| 4 | Vijverberg, 2013 | Inflammatory phenotypes underlying uncontrolled childhood asthma despite inhaled corticosteroid treatment: rationale and design of the PACMAN2 study | Study design and protocol |
| 5 | Hill, 1990 | Monitoring glucocorticoid therapy: a pharmacokinetic approach. | Direct biological measurements were done for the purpose of assessing the pharmacokinetics of oral steroids only. No adherence data were reported |
| 6 | Smy, 2016 | Hair cortisol as a novel biomarker of HPA suppression by inhaled corticosteroids in children | No biological body fluids or FeNO levels were assessed in term of adherence. |
| 7 | Zöllner, 2011 | Hypothalamic-pituitary-adrenal axis suppression in asthmatic children on inhaled and nasal corticosteroids--more common than expected? | No direct biological body fluids or FeNO levels were assessed in term of adherence. |
| 8 | Zöllner, 2012 | Hypothalamic-pituitary-adrenal axis suppression in asthmatic school children. | No direct biological body fluids or FeNO levels were assessed in term of adherence. |
| 9 | Gamble, 2011 | A study of a multi-level intervention to improve on-adherence in difficult to control asthma | No direct biological body fluids or FeNO levels were assessed in term of adherence. |
| 10 | Hagan, 2011 | Assessment of synthetic glucocorticoids in asthmatic sputum | Case report. |
| 11 | Hagan, 2011 | Analysis of fluticasone propionate in induced sputum by mass spectrometry. | Technical study to assess the detection of ICS in whole sputum, no adherence assessment were reported. |
| 12 | Hagan, 2011 | Urinary fluticasone propionate-17beta-carboxylic acid to assess asthma therapy adherence | Technical study to assess the detection of ICS in Urine. |
| 13 | Korpi-Steiner, 2010 | Liquid chromatography-tandem mass spectrometry analysis of urinary fluticasone propionate-17beta-carboxylic acid for monitoring compliance with inhaled-fluticasone propionate therapy | Technical study on non-asthmatic subjects. |
| 14 | Murphy, 2012 | The relationship between clinical outcomes and medication adherence in difficult-to-control asthma | Sputum eosinophil counts was investigated with adherence rate. |
| 15 | Papi, 2018 | Relationship of Inhaled Corticosteroid Adherence to Asthma Exacerbations in Patients with Moderate-to-Severe Asthma | Blood eosinophil counts was investigated with adherence rate. |
| 16 | Pijnenburg, 2005 | Titrating steroids on exhaled nitric oxide in children with asthma: a randomized controlled trial | No comparison between adherent and nonadherent groups and also in FeNO levels. |
| 17 | Berthon, 2015 | Effects of short-term oral corticosteroid intake on dietary intake, in adults with asthma randomized controlled trial. | Association between blood eosinophil and adherence level were tested only. |
| 18 | Shimoda, 2017 | Lung sound analysis can be an index of the control of bronchial asthma. | Lung sounds were correlated to adherence levels and FeNO. No data reported the |
| 19 | Price, 2020 | Treatment adherence in adolescents with asthma | Review article |

Table S2: results of quality assessment of included observational studies

| **Year** | **Author (ref)** | Q1 | Q2 | Q3 | Q4 | Q5 | Q 6 | Q 7 | Q 8 | Q 9 | Q 10 | Q 11 | Q 12 | Q 13 | Q 14 | QUALITY |
| --- | --- | --- | --- | --- | --- | --- | --- | --- | --- | --- | --- | --- | --- | --- | --- | --- |
| 1998 | Stirling (20) | Y | Y | Y | Y | N | CD | Y | N | N | N | Y | CD | Y | N | POOR |
| 2001 | Payne (21) | Y | Y | CD | Y | N | NA | Y | N | Y | N | Y | Y | Y | N | POOR |
| 2001 | Payne (22) | Y | Y | CD | CD | N | CD | Y | N | Y | N | Y | CD | Y | N | POOR |
| 2003 | Robinson (23) | N | Y | CD | Y | N | NA | Y | N | N | N | N | CD | Y | N | POOR |
| 2004 | Delgado (24) | Y | N | CD | Y | N | Y | Y | Y | N | Y | Y | CD | CD | Y | POOR |
| 2006 | Katsara (25) | Y | N | CD | Y | N | Y | Y | Y | Y | Y | Y | CD | Y | N | FAIR |
| 2007 | Lex (26) | Y | Y | CD | Y | N | NA | Y | N | Y | N | Y | NA | Y | N | POOR |
| 2009 | Bossley (27) | Y | Y | Y | Y | N | CD | Y | N | Y | N | Y | CD | Y | N | POOR |
| 2009 | Gamble (28) | Y | Y | Y | Y | N | Y | Y | Y | Y | Y | Y | CD | Y | Y | FAIR |
| 2010 | Cano (29) | Y | Y | Y | Y | N | N | Y | N | N | N | Y | Y | Y | Y | POOR |
| 2010 | Scott (30) | Y | N | Y | Y | N | CD | Y | N | N | N | Y | CD | NA | Y | POOR |
| 2011 | Koster (31) | Y | Y | Y | Y | N | CD | Y | N | Y | Y | Y | CD | Y | Y | FAIR |
| 2012 | Vijverberg (32) | Y | Y | CD | Y | N | NA | Y | NA | Y | NA | Y | NA | Y | Y | FAIR |
| 2012 | McNicholl (33) | Y | Y | N | NA | N | N | Y | N | CD | Y | Y | CD | Y | N | FAIR |
| 2013 | Price (34) | Y | N | CD | CD | N | Y | CD | Y | Y | Y | Y | NA | NA | N | POOR |
| 2017 | Klok (35) | Y | N | CD | Y | N | Y | Y | Y | Y | Y | Y | CD | CD | Y | POOR |
| 2017 | George (36) | Y | N | CD | Y | N | NA | Y | N | N | N | N | CD | Y | N | POOR |
| 2017 | Jochmann (37) | Y | Y | CD | Y | N | Y | Y | Y | Y | Y | Y | CD | Y | Y | GOOD |
| 2018 | Heaney (38) | Y | Y | Y | Y | N | N | Y | N | Y | Y | Y | CD | Y | N | GOOD |
| 2019 | Yuan (39) | Y | Y | CD | Y | N | Y | Y | Y | Y | Y | Y | CD | Y | Y | FAIR |
| 2020 | Vähätalo (40) | Y | N | CD | Y | N | Y | Y | Y | Y | Y | Y | CD | N | Y | FAIR |
| 2020 | Mansur (41) | Y | Y | Y | Y | N | NA | Y | Y | NA | Y | Y | CD | Y | Y | GOOD |

Abbreviation: Y: Yes; N: No; : CD: cannot determine; NA: Not applicable.

Q1. Was the research question or objective in this paper clearly stated?

Q2. Was the study population clearly specified and defined?

Q3. Was the participation rate of eligible persons at least 50%?

Q4. Were all the subjects selected or recruited from the same or similar populations (including the same time period)? Were inclusion and exclusion criteria for being in the study prespecified and applied uniformly to all participants?

Q5. Was a sample size justification, power description, or variance and effect estimates provided?

Q6. For the analyses in this paper, were the exposure(s) of interest measured prior to the outcome(s) being measured?

Q7. Was the timeframe sufficient so that one could reasonably expect to see an association between exposure and outcome if it existed?

Q8. For exposures that can vary in amount or level, did the study examine different levels of the exposure as related to the outcome (e.g., categories of exposure, or exposure measured as continuous variable)?

Q9. Were the exposure measures (independent variables) clearly defined, valid, reliable, and implemented consistently across all study participants?

Q10. Was the exposure(s) assessed more than once over time?

Q11. Were the outcome measures (dependent variables) clearly defined, valid, reliable, and implemented consistently across all study participants?

Q12. Were the outcome assessors blinded to the exposure status of participants?

Q13. Was loss to follow-up after baseline 20% or less?

Q14. Were key potential confounding variables measured and adjusted statistically for their impact on the relationship between exposure(s) and outcome(s)?

Table S3: results of quality assessment of included randomised control studies

| Year | Author | Q 1 | Q 2 | Q 3 | Q 4 | Q 5 | Q 6 | Q 7 | Q 8 | Q 9 | Q 10 | Q 11 | Q 12 | Q 13 | Q 14 | QUALITY |
| --- | --- | --- | --- | --- | --- | --- | --- | --- | --- | --- | --- | --- | --- | --- | --- | --- |
| 2002 | Beck-Ripp (42) | Y | CD | CD | N | CD | Y | Y | CD | CD | Y | Y | Y | Y | N | POOR |
| 2008 | Szefler (43) | Y | Y | Y | N | CD | CD | N | N | CD | Y | Y | Y | Y | Y | GOOD |
| 2010 | Strandbygaard (44) | N | Y | Y | N | N | Y | N | Y | Y | Y | Y | N | Y | N | POOR |
| 2020 | Koumpagioti (45) | Y | Y | CD | N | CD | Y | Y | Y | CD | Y | Y | N | Y | N | FAIR |
|  |  |  |  |  |  |  |  |  |  |  |  |  |  |  |  |  |

Abbreviations: Y: Yes; N: No; : CD: cannot determine; NA: Not applicable.

Q1. Was the study described as randomized, a randomized trial, a randomized clinical trial, or an RCT?

Q2. Was the method of randomization adequate (i.e., use of randomly generated assignment)?

Q3. Was the treatment allocation concealed (so that assignments could not be predicted)?

Q4. Were study participants and providers blinded to treatment group assignment?

Q5. Were the people assessing the outcomes blinded to the participants' group assignments?

Q6. Were the groups similar at baseline on important characteristics that could affect outcomes (e.g., demographics, risk factors, co-morbid conditions)?

Q7. Was the overall drop-out rate from the study at endpoint 20% or lower of the number allocated to treatment?

Q8. Was the differential drop-out rate (between treatment groups) at endpoint 15 percentage points or lower?

Q9. Was there high adherence to the intervention protocols for each treatment group?

Q10. Were other interventions avoided or similar in the groups (e.g., similar background treatments)?

Q11. Were outcomes assessed using valid and reliable measures, implemented consistently across all study participants?

Q12. Did the authors report that the sample size was sufficiently large to be able to detect a difference in the main outcome between groups with at least 80% power?

Q13. Were outcomes reported or subgroups analyzed prespecified (i.e., identified before analyses were conducted)?

Q14. Were all randomized participants analyzed in the group to which they were originally assigned, i.e., did they use an intention-to-treat analysis?

**References**

1. Knoery C, Brock B, Cowan J, Cox E, Taylor R. Non-adherence to inhaled corticosteroid therapy as an explanation for persistently elevated exhaled nitric oxide levels in patients with asthma: Effect of directly observed therapy with inhaled corticosteroids (DOT-ICS). *Eur Respir J*  2015;46:PA3999.
2. Agache I, Ciobanu C. Predictive value of lung function trend and FeNO for difficult asthma in children. *J Investig Allergol Clin Immunol* 2012;22:419-426.
3. Hederos CA, Janson S, Hedlin G. Six-year follow-up of an intervention to improve the management of preschool children with asthma. *Acta Paediatr* 2009;98:1939-1944.
4. Vijverberg SJ, Koenderman L, van Erp FC, van der Ent CK, Postma DS, Brinkman B, Sterk PJ, Raaijmakers JAM, Maitland-van der Zee A-H. Inflammatory phenotypes underlying uncontrolled childhood asthma despite inhaled corticosteroid treatment: rationale and design of the PACMAN2 study. *BMC Pediatr*. 2013;13:94.
5. Hill MR, Szefler SJ, Ball BD, Bartoszek M, Brenner AM. Monitoring glucocorticoid therapy: a pharmacokinetic approach. *Clin Pharmacol Ther* 1990;48:390-398.
6. Smy L, Shaw K, Smith A, Russell E, van Uum S, Rieder M, Carleton B, Koren G. Hair cortisol as a novel biomarker of HPA suppression by inhaled corticosteroids in children. *Pediatr Res* 2015;78:44-47.
7. Zöllner EW, Lombard C, Galal U, Hough S, Irusen E, Weinberg E. Hypothalamic-pituitary-adrenal axis suppression in asthmatic children on inhaled and nasal corticosteroids--more common than expected?. *J Pediatr Endocrinol Metab*. 2011;24:529-534.
8. Zöllner EW, Lombard CJ, Galal U, Hough FS, Irusen EM, Weinberg E. Hypothalamic-pituitary-adrenal axis suppression in asthmatic school children. *Pediatrics*. 2012;130:e1512-e1519.
9. Gamble J, Stevenson M, Heaney LG. A study of a multi-level intervention to improve non-adherence in difficult to control asthma. *Respir Med*. 2011;105:1308-1315.
10. Hagan JB, Taylor RL, Singh RJ. Assessment of synthetic glucocorticoids in asthmatic sputum. *Allergy Rhinol (Providence)*. 2011;2:33-35.
11. Hagan JB, Taylor RL, Kita H, Singh RJ. Analysis of fluticasone propionate in induced sputum by mass spectrometry. *Allergy Asthma Proc*. 2011;32:18-21
12. Hagan JB, Netzel BC, Matthews MR, Korpi-Steiner NL, Singh RJ. Urinary fluticasone propionate-17beta-carboxylic acid to assess asthma therapy adherence. *Allergy Asthma Proc*. 2012;33:e35-e39.
13. Korpi-Steiner NL, Netzel BC, Seegmiller JC, Hagan JB, Singh RJ. Liquid chromatography-tandem mass spectrometry analysis of urinary fluticasone propionate-17beta-carboxylic acid for monitoring compliance with inhaled-fluticasone propionate therapy. *Steroids*. 2010;75:77-82.
14. Murphy AC, Proeschal A, Brightling CE, Wardlaw AJ, Pavord I, Bradding P, Green RH. The relationship between clinical outcomes and medication adherence in difficult-to-control asthma. *Thorax* 2012;67:751-3.
15. Papi A, Ryan D, Soriano JB, Chrystyn H, Bjermer L, Rodríguez-Roisin R, Dolovich MB, Harris M, Wood L, Batsiou M, Thornhill SI, Price DB. Relationship of Inhaled Corticosteroid Adherence to Asthma Exacerbations in Patients with Moderate-to-Severe Asthma. *J Allergy Clin Immunol Pract.* 2018;6:1989-1998.e3
16. Pijnenburg MW, Bakker EM, Hop WC, De Jongste JC. Titrating steroids on exhaled nitric oxide in children with asthma: a randomized controlled trial. *Am J Respir Crit Care Med*. 2005;172:831-836
17. Berthon BS, Gibson PG, McElduff P, MacDonald-Wicks LK, Wood LG. Effects of short-term oral corticosteroid intake on dietary intake, body weight and body composition in adults with asthma - a randomized controlled trial. *Clin Exp Allergy*. 2015;45:908-919.
18. Shimoda T, Obase Y, Nagasaka Y, Nakano H, Kishikawa R, Iwanaga T. Lung sound analysis can be an index of the control of bronchial asthma. *Allergol Int*. 2017;66:64-69.
19. Kaplan A, Price D. Treatment Adherence in Adolescents with Asthma. *J Asthma Allergy*. 2020;13:39-49.
20. Stirling RG, Kharitonov SA, Campbell D, Robinson DS, Durham SR, Chung KF, et al. Increase in exhaled nitric oxide levels in patients with difficult asthma and correlation with symptoms and disease severity despite treatment with oral and inhaled corticosteroids. Thorax 1998;53:1030-4.
21. Payne DN, Wilson NM, James A, Hablas H, Agrafioti C, Bush A. Evidence for different subgroups of difficult asthma in children. Thorax 2001;56:345-50
22. Payne DN, Adcock IM, Wilson NM, Oates T, Scallan M, Bush A. Relationship between exhaled nitric oxide and mucosal eosinophilic inflammation in children with difficult asthma, after treatment with oral prednisolone. Am J Respir Crit Care Med 2001;164:1376-81.
23. Robinson DS, Campbell DA, Durham SR, Pfeffer J, Barnes PJ, Chung KF. Systematic assessment of difficult-to-treat asthma. Eur Respir J 2003;22:478-83.
24. Delgado-Corcoran C, Kissoon N, Murphy SP, Duckworth LJ. Exhaled nitric oxide reflects asthma severity and asthma control. Pediatric Crit Care Med 2004;5:48-52.
25. Katsara M, Donnelly D, Iqbal S, Elliott T, Everard ML. Relationship between exhaled nitric oxide levels and compliance with inhaled corticosteroids in asthmatic children. Respir Med 2006;100:1512-7.
26. Lex C, Jenkins G, Wilson NM, Zacharasiewicz A, Erin E, Hansel TT, et al. Does sputum eosinophilia predict the response to systemic corticosteroids in children with difficult asthma? Pediatric Pulmonol 2007;42:298-303.
27. Bossley CJ, Saglani S, Kavanagh C, Payne DN, Wilson N, Tsartsali L, et al. Corticosteroid responsiveness and clinical characteristics in childhood difficult asthma. Eur Respir J 2009;34:1052-9.
28. Gamble J, Stevenson M, McClean E, Heaney LG. The prevalence of nonadherence in difficult asthma. Am J Respir Crit Care Med 2009;180:817-22.
29. Cano-Garcinuno A, Carvajal-Uruena I, Diaz-Vazquez CA, Dominguez-Aurrecoechea B, Garcia-Merino A, de Rodas PMC, et al. Clinical Correlates and Determinants of Airway Inflammation in Pediatric Asthma. J Investig Allergol Clin Immunol 2010;20:303-10.
30. Scott M, Raza A, Karmaus W, Mitchell F, Grundy J, Kurukulaaratchy RJ, et al. Influence of atopy and asthma on exhaled nitric oxide in an unselected birth cohort study. Thorax 2010;65:258-62.
31. Koster ES, Raaijmakers JA, Vijverberg SJ, Maitland-van der Zee AH. Inhaled corticosteroid adherence in paediatric patients: the PACMAN cohort study. Pharmacoepidemiol Drug Saf. 2011;20:1064-72.
32. Vijverberg SJ, Koster ES, Koenderman L, Arets HG, van der Ent CK, Postma DS, et al. Exhaled NO is a poor marker of asthma control in children with a reported use of asthma medication: a pharmacy-based study. Pediatric Allergy Immunol 2012;23:529-36.
33. McNicholl DM, Stevenson M, McGarvey LP, Heaney LG. The utility of fractional exhaled nitric oxide suppression in the identification of nonadherence in difficult asthma. Am J Respir Crit Care Med 2012;186:1102-8
34. Price D, Ryan D, Burden A, Von Ziegenweidt J, Gould S, Freeman D, et al. Using fractional exhaled nitric oxide (FeNO) to diagnose steroid-responsive disease and guide asthma management in routine care. Clin Translational Allergy 2013;3:37.
35. Klok T, Brand PLP. Can exhaled nitric oxide fraction predict adherence to inhaled corticosteroids in atopic and nonatopic children with asthma? J Allergy Clin Immunol in practice 2017;5:521-2.
36. George KE, Ryan DM, Keevil B, Niven R, Fowler SJ. A pilot study to investigate the use of serum inhaled corticosteroid concentration as a potential marker of treatment adherence in severe asthma. J Allergy Clin Immunol 2017;139:1037-9.
37. Jochmann A, Artusio L, Jamalzadeh A, Nagakumar P, Delgado-Eckert E, Saglani S, et al. Electronic monitoring of adherence to inhaled corticosteroids: an essential tool in identifying severe asthma in children. Eur Respir J. 2017;50:1700910.
38. Heaney LG, Busby J, Bradding P, Chaudhuri R, Mansur AH, Niven R, et al. Remotely Monitored Therapy and Nitric Oxide Suppression Identifies Non-Adherence in Severe Asthma. Am J Resp Crit Care Med. 2019;199:454-464.
39. Yuan Y, Li B, Huang M, Peng X, Zhao W, Ye Y, et al. Fractional exhaled nitric oxide was not associated with the future risk of exacerbations in Chinese asthmatics: a non-interventional 1-year real-world study. J Thoracic Dis 2019;11:2438.
40. Vähätalo I, Ilmarinen P, Tuomisto LE, Tommola M, Niemelä O, Lehtimäki L, et al. 12-year adherence to inhaled corticosteroids in adult-onset asthma. Eur Respir J Open Res 2020;6.
41. Mansur AH, Hassan M, Duffy J, Webster C. Development and clinical application of a prednisolone/cortisol assay to determine adherence to maintenance oral prednisolone in severe asthma. Chest 2020 in press; doi: 10.1016/j.chest.2020.03.056.
42. Beck-Ripp J, Griese M, Arenz S, Koring C, Pasqualoni B, Bufler P. Changes of exhaled nitric oxide during steroid treatment of childhood asthma. Eur Respir J 2002;19:1015-9.
43. Szefler SJ, Mitchell H, Sorkness CA, Gergen PJ, O'Connor GT, Morgan WJ, et al. Management of asthma based on exhaled nitric oxide in addition to guideline-based treatment for inner-city adolescents and young adults: a randomised controlled trial. Lancet 2008;372:1065-72.
44. Strandbygaard U, Thomsen SF, Backer V. A daily SMS reminder increases adherence to asthma treatment: a three-month follow-up study. Respir Med 2010;104:166-71.
45. Koumpagioti D, Boutopoulou B, Priftis KN, Douros K. Effectiveness of an educational program for children and their families on asthma control treatment adherence. J Asthma 2020;57:567-73.
